# Exercise Capacity in Unilateral Diaphragm Paralysis: The Effect of Obesity

**DOI:** 10.1155/2019/1090982

**Published:** 2019-04-01

**Authors:** Paul S. Richman, Pomin Yeung, Thomas V. Bilfinger, Jie Yang, William W. Stringer

**Affiliations:** ^1^Pulmonary and Critical Care Division, Stony Brook University Health Sciences Center, Stony Brook, NY 11794-8172, USA; ^2^Weill Cornell University Medical Center, 1300 York Ave, New York, NY 10065, USA; ^3^Cardiothoracic Surgery Division, Stony Brook University Health Sciences Center, Stony Brook, NY 11794-8172, USA; ^4^Department of Family, Population & Preventive Medicine, Stony Brook University Health Sciences Center, Stony Brook, NY 11794-8172, USA; ^5^Los Angeles Biomedical Institute (LABIOMED) at Harbor-UCLA Medical Center, 1000 West Carson St, Torrance, CA 90509, USA

## Abstract

**Purpose:**

Healthy patients with unilateral diaphragm paralysis (UDP) are often asymptomatic; those with UDP and comorbidities that increase work of breathing are often dyspneic. We report the effect of obesity on exercise capacity in UDP patients.

**Methods:**

All obese and nonobese patients with UDP undergoing cardiopulmonary exercise testing (CPET) during a 32-month period in the exercise laboratory of an academic hospital were compared to a retrospectively identified cohort of obese and nonobese controls without UDP, matched for key features. CPET used a modified Bruce treadmill protocol with breath-to-breath expired gas analysis. O2 uptake, minute ventilation, exercise time, and work rate were recorded at peak exercise. Static pulmonary functions were measured. Kruskal-Wallis, Wilcoxon rank sum, and Fisher's exact tests were used to compare continuous and categorical variables, respectively. Stratified linear regression was used to quantify the effect of UDP and obesity on CPET variables.

**Results:**

Twenty-two UDP patients and 46 controls were studied. The BMI of obese and nonobese patients was 33.0±4.2 and 25.8±2.4 kg/m2, respectively. UDP subjects with obesity, compared to controls with neither condition, showed significantly reduced peak O2 uptake normalized to actual body weight (1.57±0.64 versus 2.01±0.88 L/min), shorter exercise time (5.7±2.0 versus 8.5±2.9 minutes), and lower peak ventilation. This was not observed in UDP alone or obesity alone. Peak work rate trended lower in the combined UDP-obesity group.

**Conclusion:**

Neither UDP nor obesity alone significantly reduced exercise capacity. Superimposed UDP and obesity interact to create a ventilatory limitation to exercise, with reduced peak-VO2, exercise time, and work rate.

## 1. Introduction

Patients with unilateral paralysis of the diaphragm (UDP) have near-normal static pulmonary function and are only mildly dyspneic during exercise, if there is no coexisting cardiopulmonary disease [1–3]. Cardiopulmonary exercise testing (CPET) in healthy subjects with UDP has shown mild or insignificant reduction in peak O2 uptake (VO2peak) and only mildly reduced total exercise time [2, 4], unlike those with bilateral diaphragm paralysis who have significant exercise limitation [2, 5]. However, many patients with UDP have comorbid disease of the heart or lungs; dyspnea on exertion has been commonly reported in such patients [1, 6, 7]. Thus, it seems likely that exercise limitation in UDP patients is magnified by any coexisting disorder that increases the work of breathing, such as chronic hyperinflation, V/Q mismatch, or poor compliance of the respiratory system.

Obesity is a common disorder that imposes a mechanical load during exercise, limiting the capacity to do external work. Prior studies have shown that peak work rate during cycle ergometry is reduced in healthy moderately obese subjects [8–12], despite generally normal values for peak VO2, O2 pulse, and anaerobic threshold (AT). This is referred to as mechanical inefficiency: O2 consumption is relatively high at any given level of external work, reflecting an excessive metabolic cost of moving heavy limbs [10, 12]. In addition, inertia and noncompliance of the obese chest wall may impose an intolerable load on the respiratory muscles causing dyspnea that limits exercise capacity [13].

We postulate that the generally mild exercise limitation among UDP patients is magnified by these mechanical loads due to obesity. No studies have specifically addressed this issue. The aim of this study was to determine the combined effects of obesity and UDP on exercise limitation in a cohort of patients undergoing CPET in our exercise laboratory.

## 2. Methods

### 2.1. Study Design

**Figure 1:**
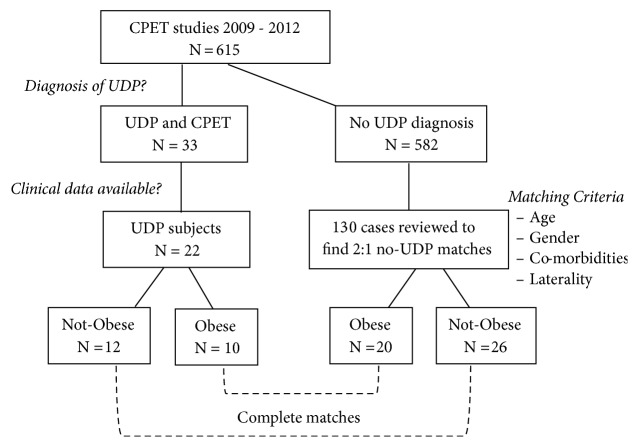
Flow diagram of the study.

This was a matched-cohort study of patients with a confirmed diagnosis of UDP in the pulmonary practice of an academic medical center who performed CPET during a 32-month period from 2009 to 2013. Comorbidities were recorded from each subject's chart and placed in four disease categories: cardiovascular, pulmonary, neuromuscular, or joint/pain disorder. The definition of each type of comorbidity is detailed in the Supplementary Materials (available here). UDP subjects were categorized as obese (body mass index, BMI, ≥30) or nonobese (BMI<30). We matched each obese and nonobese UDP subject to 2 control subjects without UDP who underwent CPET in that timeframe. Controls were screened in batches of 10 until at least two matching controls were identified for every UDP patient. All screened controls were included; as a result, in two cases a UDP subject was matched to 3 controls. Eight matching criteria were utilized: age within 10 years, gender, presence of obesity, laterality of UDP, and presence of each of the above 4 comorbidities. This resulted in 4 distinct study subgroups: UDP/obese; UDP/nonobese; no-UDP/obese; no-UDP/nonobese (flow diagram, Figure 1).

### 2.2. Diagnosis of Unilateral Diaphragm Paralysis

UDP patients had all of the following: diminished unilateral breath sounds, PA and lateral upright chest X-ray demonstrating asymmetric elevation of one hemidiaphragm, and diaphragm fluoroscopy (“sniff testing”) showing paradoxical upward motion of one hemidiaphragm [14]. We included only cases with paradoxical motion of a hemidiaphragm, not those with diminished or asynchronous descent of a hemidiaphragm, which may reflect only weakness or eventration. The sniff test was performed within 2 months prior to CPET. Duration of UDP at the time of CPET testing was estimated by the onset-time of symptoms, the date of the probable causative event (e.g., surgery) and prior X-rays.

### 2.3. Pulmonary Function (PFT) Tests

Subjects were tested in the sitting position (KoKo Px, NSpire Health, Longmont, CO, USA) with recording of maximal FEV1 and FVC during 3 forced expiratory maneuvers, following ATS/ERS guidelines [15]. The majority of patients had spirometry repeated while supine. NHANES-III reference equations [16] were used to calculate FVC as percent predicted (FVC %pred). Maximal inspiratory pressure (MIP) at RV and maximal expiratory pressure (MEP) at TLC were determined in the sitting position using a hand-held manometer (Instrumentation Industries Inc., Bethel Park, PA, USA).

### 2.4. Exercise Testing

Subjects performed symptom-limited exercise on an incremental treadmill protocol (modified Bruce protocol) with breath-to-breath analysis of minute ventilation (VE), O2 consumption, and CO2 excretion (Vmax Encore CPET System, Becton, Dickinson, USA). O2-hemoglobin % saturation by pulse oximetry and EKG tracings (CardioSoft, G.E. Healthcare, Chicago IL, USA) were measured continuously and recorded each minute. The duration of exercise and reason for stopping exercise were recorded. Predicted maximal O2 uptake was calculated by the equations: Women: VO_2_ max/kg =* *42.83– (0.371 *∗* years). Men: VO_2_ max/kg =* *50.02– (0.394 *∗* years) [11]. The equation of Jones [17] was used to estimate work rate during treadmill exercise: peak work rate (PWR, watts) = 9.81(m*∗*v*∗*i)/100, where m is mass (kg), v is velocity(m/sec), and i is %incline of the treadmill.

### 2.5. Statistical Analysis

The Kruskal-Wallis test was used to compare continuous clinical variables among the four groups. Wilcoxon rank sum test was used to compare the continuous demographic and clinical variables between obese DP and nonobese DP groups. Fisher's exact tests were used to compare categorical variables between obese DP and nonobese DP groups. To quantify the effect of obesity and UDP on exercise performance, a stratified linear regression was used to test the association of UDP, obesity, or their combination on six CPET variables: peak VO2 expressed as % predicted by actual and ideal body weight (VO2Max% ABW and VO2Max% IBW, respectively), Peak VE, breathing reserve, estimated PWR, and exercise time; the strength of the association is expressed by their estimated coefficients. Obesity (yes vs. no), UDP (yes vs. no), and their interaction were considered as explanatory variables. Statistical significance was set at 0.05. Analysis was done using SAS 9.3 (SAS Institute Inc., Cary, NC).

### 2.6. Human Subjects Protection

This study was conducted in accordance with the amended Declaration of Helsinki. The study was approved by Stony Brook University's Committee on Research Involving Human Subjects (CORIHS approval # 207533-1). It was exempted from the requirement of informed consent, because of its low-risk and deidentified data collection.

## 3. Results

### 3.1. Subject Characteristics

The study included 68 subjects in four groups as noted above. 22 subjects had UDP; 46 matched controls did not. By design of the matching process, there were no significant differences between UDP and control subjects with respect to demographic and clinical features (Table 1). The difference in BMI between obese and nonobese subjects was statistically significant and clinically meaningful: 33.3 +/- 4.2 kg/m2 versus 25.8 +/- 2.4 kg/m2 (mean +/- SD). Our obese subjects tended to be more female and have fewer cardiac and pulmonary comorbidities (nonsignificant differences).

### 3.2. PFTs and CPET Parameters (Table 2 and Figures 2(c) and 2(d))

**Figure 2:**
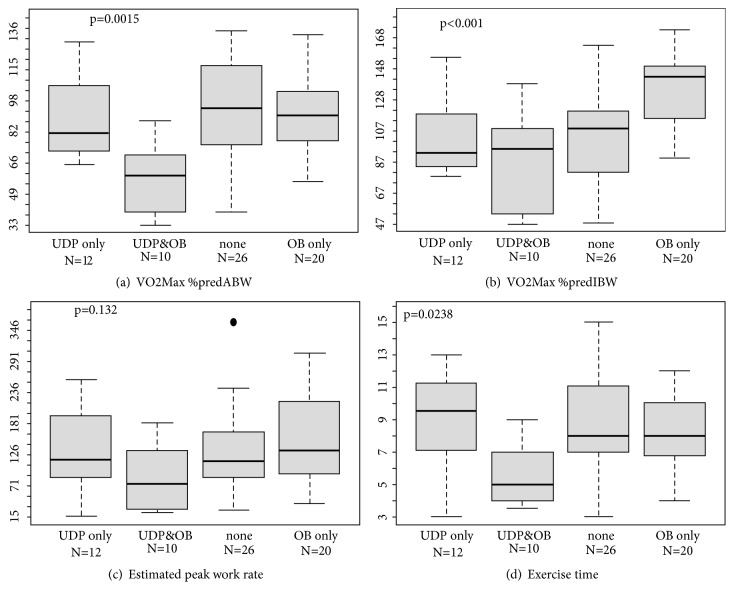
CPET parameters in matched UDP and non-UDP subjects, with and without obesity. Box plot showing the interquartile range, median, min, max, and possible outliers. UDP= unilateral diaphragm paralysis; OB= obesity. Outliers are indicated as dots on the figure. p-values based on stratified linear regression. Panel (a): VO2 at peak exercise, as percent predicted referenced to* actual* body weight. Panel (b): VO2 at peak exercise, as percent predicted referenced to* ideal* body weight. Panel (c): (PWR, watts) = 9.81(m*∗*v*∗*i)/100, where m is mass (kg); v is velocity (m/sec); i is treadmill %incline. Panel (d): total exercise time, minutes.

Obesity alone was not associated with a restrictive PFT pattern (though FRC was reduced) and no decline in peak minute ventilation. The absolute value for peak O2 uptake and estimated peak work rate were observed to reach supranormal values in subjects with obesity alone. Conversely, UDP alone was associated with mild restriction, a trend to lower peak VE (coefficient estimate 16.9, p=0.06), absence of a breathing reserve, and slightly reduced absolute value for peak O2 uptake (p=.04). However, exercise capacity was not affected by UDP alone, as shown by preserved values for peak work rate and exercise time. The combination of obesity and UDP was associated with more pronounced restriction, a 29% reduction in peak minute ventilation, and a breathing reserve that was <12% of the predicted maximal VE, suggesting a respiratory limitation compared to non-UDP controls. With regard to exercise capacity, the regression model showed that UDP lowered exercise time significantly only for obese subjects, not for nonobese subjects. There was a similar trend for a combined effect of UDP and obesity on PWR, which did not reach statistical significance (p=0.13) (see Figures 2(c) and 2(d)).

### 3.3. Oxygen Uptake and Work Efficiency

Peak VO2 expressed as % predicted for* actual* body weight (VO2_Max_% ABW) was significantly lower in the group with combined UDP and obesity compared to the other 3 groups (Figure 2(a), coefficient estimate: −28.2, p=.001). Peak VO2 expressed as % predicted for* ideal* body weight (VO2_Max_% IBW) was higher in the group with obesity alone, compared to the other 3 groups (Figure 2(b), coefficient estimate: 31.7, p<.001). The regression model showed that VO2_Max_% IBW was reduced by UDP only in obese subjects, not in normal-weight subjects.

We estimated work efficiency at peak exercise, defined as peak work rate/peak-VO2. This variable was not affected by either UDP or obesity alone. However, subjects with combined obesity and UDP showed a trend toward lower exercise efficiency (p=0.12).

### 3.4. Oxygenation

All groups showed a small average decline in SpO2 at peak exercise, although <90% in only 7 subjects. Eight subjects experienced a >5% drop in SpO2. These subjects, compared to others, had a higher prevalence of concomitant cardiovascular disease (88% vs. 38%) and lower O2 pulse at peak exercise (10.9 vs. 16.0 ml/beat), though prevalence of concomitant respiratory disease and spirometric values were similar. DLCO was not substantially different between the groups (Table 2).

### 3.5. Other Findings

Thirty-eight of the 46 subjects achieved an anaerobic threshold (Table 2). The anaerobic threshold was normal (mean value >55% of predicted VO2-max) in all groups except the combined UDP- Obese group, which was mildly reduced at 44%. A subset of 12 UDP subjects performed both sitting and supine spirometry; the mean decline in FVC (sitting to supine) was 26%. Their mean maximal inspiratory and expiratory pressures (MIP and MEP) were -55 and +84 cmH2O, respectively. There was no between-group difference in FEV-1/FVC ratio.

## 4. Discussion

The main findings of our study are that obese subjects with UDP have markedly reduced peak O2 uptake when normalized to actual body weight, a lower peak minute ventilation, and exercise for a shorter time on an incremental treadmill protocol. Subjects with obesity alone or UDP alone did not show this reduction in peak VO2. The interactive effect of UDP and obesity on peak O2 uptake was evident whether VO2 is expressed in relation to the patient's actual or ideal body weight. Consistent with these findings, we also found a trend for a lower estimated peak work rate in the group with combined UDP and obesity. This would appear to support the idea that a subtle exercise limitation in UDP is magnified by both the excessive metabolic cost of moving heavy limbs and the inertia and noncompliance of the obese chest wall. To our knowledge this is the first study to quantify their interactive effect on exercise performance, though there is a large body of prior related research.

### 4.1. Effect of Obesity Alone on Exercise Performance

It is well demonstrated that during cycle ergometry, obese subjects compared to lean ones have a higher VO2 at each incremental work rate, even though the slope of the VO2/WR curve is normal, indicating reduced mechanical exercise efficiency [10, 18, 19]. At any given level of submaximal exercise, minute ventilation is also higher among obese compared to lean individuals, indicating a normal ventilatory response to the high metabolic demand of exercising with a heavy body [20, 21]. At peak exercise, multiple studies have shown that healthy obese subjects achieve maximal work rates that are 9%-28% lower than lean controls, due to the excessive metabolic demand of moving heavy limbs [10, 19, 21–23] and ventilating a heavy chest wall [13, 21]. These same studies have also shown overall equivalent values for VO2 at peak exercise (approximately 2.3 L/min) as well as equal VO2 at the ventilatory threshold, in both obese and lean subjects. These numerical data are derived from healthy individuals with moderate-to-severe obesity (BMI ~40) and indicate preserved aerobic function of the cardiorespiratory system in obesity with normal supply of O2 to the exercising muscles and normal ventilatory drive, even though peak external work capacity is reduced. This differs somewhat from the findings in our subjects, most of whom had concurrent cardiopulmonary disease and were less obese: our obese subjects without UDP had BMI ranging from 31 to 38 and exercised to PWR values that were actually a bit higher than nonobese subjects, though they achieved this PWR at a value of peak VO2 that was substantially higher than that of nonobese subjects, when indexed to ideal body weight. This implies aerobic “conditioning” was greater in the obese compared to nonobese subjects, most likely a training effect induced by the high energy-cost of daily activities [22, 24, 25]. The relatively milder obesity in our subjects may account for this discrepancy between our study and other published work, since the degree of obesity is known to affect exercise capacity, fitness, and work efficiency.

### 4.2. Effect of Combined Obesity and UDP on Exercise Performance

Both groups of UDP subjects (obese and nonobese) had low values for FVC and peak minute ventilation and essentially had no ventilatory reserve. Sixteen of the 22 UDP subjects (73%) adopted a rapid, shallow breathing pattern at peak exercise and thus a high dead-space fraction that may have reduced alveolar ventilation even further. This ventilatory limitation did not reduce the peak work rate or exercise time among nonobese subjects. Obese subjects, however, also breathe with lower tidal volume and higher frequency during exercise, most likely to reduce elastic work of breathing [26]. When this effect is superimposed on the rapid-shallow respiratory pattern of UDP, it is reasonable to infer that the work of breathing become intolerable. In this case, the obese subject with UDP reaches a ventilatory limitation to exercise that imposes a “ceiling” limit on the supranormal level of VO2 that is required to sustain the high metabolic cost of moving heavy limbs and ventilating a heavy chest wall. Thus, in our study, while neither obesity nor UDP alone reduced peak work rate, the combined effect of high metabolic burden due to obesity and ventilatory limitation due to UDP was associated with a substantial reduction in peak work rate and exercise time on an incremental protocol. Notably, the normalization of VO2 to actual body weight (not ideal body weight) was a good indicator of reduced exercise capacity due to the interaction of UDP and obesity (Figure 2(a)). Conversely the normalization of peak VO2 relative to ideal body weight was a better indicator of the “conditioning” effect of obesity (Figure 2(b)).

Small declines in SpO2 at peak exercise were seen equally in all groups, generally in those with underlying cardiac disease. Its severity was not associated with obesity or UDP.

### 4.3. Methodologic Limitations of the Study

First, this is a retrospective study; a substantial number of potential subjects were rejected due to missing clinical data. Second, half our subjects had concurrent cardiorespiratory disease. This potentially confounds our observations on the effects of obesity and UDP on exercise capacity, despite the matching process. However, our findings are relevant to the real-world clinical situation, where obesity coexists with cardiopulmonary comorbidities. Third, among the* non*obese groups, mean BMI was 25.8, slightly above normal (i.e., roughly half were overweight, though not obese), reducing the BMI-separation between groups. These two factors may account for our finding that PWR in our nonobese subjects was approximately 25% lower than the values reported by others in lean healthy subjects. Nonetheless we demonstrated a difference in exercise performance related to obesity. The use of BMI to describe obesity is itself problematic. Individuals with identical BMI have differing amounts and/or distribution of body fat, resulting in variable mechanical loads on the respiratory muscles. In this retrospective analysis we did not quantify body fat or truncal obesity, variables known to determine how obesity impacts respiratory function [27]. Fourth, calculating peak work rate on treadmill testing by a standard formula is controversial, since it does not fully account for the energy expended accelerating and decelerating the limbs. The metabolic cost of exercise in obesity is magnified in weight-bearing (treadmill) protocols compared to cycle ergometry, though treadmill testing tends to elicit higher PWR and peak VO2 values [18, 28, 29]. These studies, which used the same equation to calculate treadmill PWR, found that at comparable work rates, treadmill and cycle modalities yield similar data for exercise time, dyspnea scores, lung mechanics, and ventilatory equivalents, supporting use of this equation. In this regard, the calculated peak work rates in our non-UDP subjects were almost identical to the mean published values of PWR by cycle ergometry (149.1 W versus 148.8 W). Finally, fluoroscopic sniff testing was used to document UDP in our cohort. Its accuracy in diagnosing UDP has been questioned, with reports of false-negatives and false-positives [14, 30]. When correctly performed, however, fluoroscopy demonstrates paradoxical inspiratory elevation in >90% of cases with phrenic nerve paralysis [7]. It has shown 81% concordance with ultrasonic assessment of diaphragm function, another widely used method [31, 32].

### 4.4. Conclusion and Clinical Implications

Normal-weight patients with UDP have a mildly reduced peak minute ventilation but normal exercise capacity. Moderate obesity alone does not significantly reduce exercise performance. Superimposed UDP and obesity create a substantial ventilatory limitation to exercise, with reductions in peak-VO2 and peak work rate. Patients who are dyspneic in association with UDP should be cautioned against gaining weight. In already obese UDP patients, the effect of weight reduction or surgical treatment of diaphragm dysfunction deserves further study.

## Figures and Tables

**Table 1:** Demographic matching of UDP to non-UDP subjects.

	Obese		Non-Obese		
	UDP (n=10)	No-UDP (n=20)	UDP (n=12)	No-UDP (n=26)	P-value
Age, years (mean +/- SD)	59 +/- 11	60 +/- 6.4	64 +/- 9	61 +/- 11	P = 0.31

Male Gender, n (%)	6 (60)	12 (60)	11 (92)	22 (92)	P = 0.14

Right-Sided (proportion)	5/10	-	7/12	-	NS

Duration UDP (mean yrs)	2.8 +/- 1.6	-	2.2 +/- 1.3	-	NS

BMI (mean +/- SD)	34.2 +/- 6.3	33.0 +/- 2.7	26.9 +/- 1.5	25.4 +/- 2.6	<.0001^*∗*^

Comorbidities, n (%)					

Cardiovascular	3 (30)	6 (30)	6 (50)	14 (54)	P = 0.41

Pulmonary	3 (30)	6 (30)	6 (50)	14 (54)	P = 0.41

Neuromuscular	0 (0)	0 (0)	0 (0)	0 (0)	-

Joint/Pain syndrome	1 (10)	2 (10)	1 (8)	2 (8)	P = 1.0

Numerical values are reported as mean +/- standard deviation.

UDP = unilateral diaphragm paralysis.

^*∗*^For the difference between obese and nonobese groups, using Wilcoxon rank-sum test and Fisher's exact test as described in the Methods.

**Table 2:** Effect of UDP, obesity, and their combination on static PFTs and exercise parameters.

	Not obese	Obese	UDP + Not obese	UDP + Obese	p-value^*∗*^
**Static PFT values**					

FEV-1, absolute value Liters	2.57 +/- 0.93	2.74 +/- 0.65	1.96 +/- 0.80	1.91 +/- 0.58	p=.004

Forced vital capacity % predicted	91 +/- 17	99 +/- 12	67 +/- 17	62.9 +/- 13.5	p<.0001

Total lung capacity % predicted	97 +/- 18	94 +/- 15	74 +/- 14	66.1 +/- 14.0	p=.0002

Functional residual capacity % predicted	99 +/- 21	73 +/- 18	71 +/- 15	53.7 +/- 12.0	p<.0001

DLCO/Alveolar volume % predicted	84 +/- 26	98 +/- 16	103 +/- 14	111.7 +/- 20.3	p=.054

**Exercise parameters**					

O2 uptake, peak exercise (VO2_peak_ L/min)	2.01 +/- 0.88	2.33 +/- 0.67	1.85 +/- 0.83	1.57 +/- 0.64	p=.04

Peak minute ventilation, VE (L/min)	76.4 +/- 35.3	78.4 +/- 21.0	66.2 +/- 24.0	54.4 +/- 23.2	p=.06

Breathing Reserve % of predicted VE (max)^*ǂ*^	16.1 +/- 22.0	15.4 +/- 18.4	-0.81 +/- 21.5	11.9 +/- 19.5	p=.16

Estimated peak work rate (PWR, watts)	132.4 +/- 76.6	149 +/- 74.7	130 +/- 75.8	93.9 +/- 66.2	p=.13

Total exercise time (minutes)	8.5 +/- 2.9	8.1 +/- 2.2	8.9 +/- 3.1	5.7 +/- 2.0	p=.008

Estimated work efficiency at peak exercise (watts/L/min)^§^	64.7 +/- 23.5	61.8 +/- 20.7	68.2 +/- 27.1	51.4 +/- 21.2	p=.23

Anaerobic Threshold^*Ϯ*^ % of maximal predicted VO2	64 +/- 18	62 +/- 14	59 +/- 13	44 +/- 17	p=.03

SpO2, resting minus nadir (%)	3.0 +/- 3.6	2.0 +/- 1.9	4.3 +/- 3.3	2.9 +/- 3.8	p=.61

Numerical values are reported as mean +/- standard deviation.

UDP = unilateral diaphragm paralysis.

^*∗*^p-values are based on the Kruskal-Wallis test.

^§^Estimated peak work efficiency is calculated as the ratio PWR/VO2peak.

^*ǂ*^Breathing reserve is expressed as the percent reduction in peak VE below the predicted maximal VE.

^*Ϯ*^No clear anaerobic threshold could be identified in 8 of the 46 subjects.

## Data Availability

The PFT data, cardiopulmonary exercise data, and clinical data used to support the findings of this study are available from the corresponding author upon request.

## Abbreviations

BMI: Body mass index
CPET: Cardiopulmonary exercise testing
OB: Obese
PWR: Peak work rate
UDP: Unilateral diaphragm paralysis
VE: Minute ventilation
VO2peak: Oxygen uptake at peak exercise
VO2Max% ABW: Maximal oxygen uptake as a percent predicted for actual body weight
VO2Max% IBW: Maximal oxygen uptake as a percent predicted for ideal body weight.

## Supplementary Materials

Supplementary Materials This section provides clinical details for the 4 types of comorbidities present in our subjects: cardiovascular, pulmonary, neuromuscular, or joint/pain disorders.
