# Supplementary material for: Exercise Capacity in Unilateral Diaphragm Paralysis: The Effect of Obesity
Source: Pulm Med. 2019 Apr 1;2019:1090982. doi: 10.1155/2019/1090982 (PMC6463570; doi:10.1155/2019/1090982)
Supplement: Supplementary Materials — This section provides clinical details for the 4 types of comorbidities present in our subjects: cardiovascular, pulmonary, neuromuscular, or joint/pain disorders. [file 1090982.f1.docx]

**Appendix**

**DEFINITIONS OF COMORBIDITIES**

CARDIOVASCULAR:

Congestive heart failure, with symptoms, either reduced or preserved ejection fraction.

Coronary artery disease (stress testing or catheterization) medically or surgically treated.

Persistent or recurrent arrhythmia.

Peripheral vascular disease, resulting in leg ischemia or vascular bypass surgery

Chronic anemia, Hgb <8.0

RESPIRATORY

Any obstructive airway disease with symptoms and FEV-1/FVC <0.7

Interstitial lung disorder with FVC <80% predicted or DLCO <70% predicted

Pulmonary hypertension of any cause with report of shortness of breath

Recurrent pneumonia or bronchitis and report of shortness of breath.

Pleural effusion (non-cardiac) requiring recent thoracentesis.

Major thoracic trauma by history

NEUROMUSCULAR:

History of stroke or traumatic brain injury

Any neuromyopathy causing weakness or limitation of daily activities

Parkinson’s disease

Peripheral neuropathy requiring treatment

Ataxia of any etiology

Weakness or >10 Kg weight loss due to cancer

JOINT/PAIN

Chronic arthropathy with symptoms

Joint fusion surgery

Poorly-healed fracture

Chronic pain syndrome, any etiology

Chronic muscle spasms
